# Molecular docking analysis of Clostridium perfringens beta toxin model with potential inhibitors from the ZINC database

**DOI:** 10.6026/97320630017628

**Published:** 2021-06-30

**Authors:** Amit Kumar Solanki, Abhishek Acharya, Himani Kaushik, Bharti Bhatia, Lalit C Garg

**Affiliations:** 1 Gene Regulation Laboratory, National Institute of Immunology, Aruna Asaf Ali Marg, New Delhi - 110067, India

**Keywords:** Clostridium perfringens, beta-toxin, molecular docking, necrotic enteritis, virtual screening, ZINC database

## Abstract

Beta toxin from Clostridium perfringens after being secreted in gut is capable of causing necrotic enteritis in humans and several other animal species and does not respond to routinely used antibiotics. Therefore, there is a need to design an effective inhibitor for the Clostridium perfringens beta toxin (CPB) using cutting edge drug discovery technologies. Hence, potential CPB inhibitors were identified using computer aided screening of compounds from the ZINC database. Further, we document the molecular docking analysis of Clostridium perfringens beta toxin model (that revealed 4 binding pockets, A-D) with the identified potential inhibitors. We show that ZINC291192 [N-[(1-methylindol-3-yl) methyl eneamino]-7,10-dioxabicyclo[4.4.0]deca-2,4,11-triene-8- carboxamide] has optimal binding features with calculated binding energy of -10.38 kcal/mol and inhibition constant of 24.76 nM for further consideration.

## Background

Clostridium perfringens responsible for necrotizing enteritis is an opportunistic pathogen possessing more than 16 various toxins and are capable of causing wide range of histotoxicity and intestinal infections [1]. C. perfringens type C secretes alpha toxin (CPA) and beta toxin (CPB) along with perfringolysin O (PFO), beta2 toxin (CPB2) and/or enterotoxin (CPE) [2]. It is known that CPB is both required and sufficient for causing virulence [2,3]. CPB is a 34,861 Da protein comprising of 309 amino acids and shares sequence similarity with H-pore-forming toxin family (delta-toxin, Net-B toxin, alpha-toxin, leukocidin & gamma-toxin) [4]. The released toxin by itself is capable of causing toxinosis even if the bacteria have died or have cleared from host. Primary treatments for type C infection include administration of broad-spectrum antibiotics such as penicillin, erythromycin and nitrofurantoin [5], but are of little value after onset of symptoms, which are mainly due to toxin and not bacteria. CPB is active as monomer and readily forms oligomeric complex on primary porcine and human endothelial cells, as well as monocytes in vitro [6]. It has also been shown that methyl-H-cyclodextrin (MHCD) can reduce the binding of the toxin to lipid rafts and swelling induced by the toxin via encapsulating the membrane cholesterol in vitro [7]. However, treatment of the cell membranes with nystatin shows that the toxin does not directly interact with cholesterol [8]. The inhibitory effect of MHCD or cholesterol oxidase thus appears to be due to changes in the properties of lipid rafts that occur when cholesterol is removed from lipid rafts by MHCD and cholesterol oxidase [6,9]. Homology modelled structures of the CPB and its receptor P2X7 have been used to identify the amino acid residues involved in their interaction [10]. With no other reports on potential drugs that directly inactivate CPB, it is of interest to identify and document potential inhibitors interacting with the CPB by molecular docking analysis of the homology modelled CPB structure.

## Methodology

### Homology modeling of CPB:

The structure for CPB was modeled on the structure of Clostridium perfringens delta-toxin [PDB ID: 2YGT]. Automated modeling method was employed for this purpose, using the SWISS-MODEL web interface [11]. Herein, the SWISS-MODEL template library (SMTL version 2016-05-26, PDB release 2016-05-20) was searched with BLAST [12] and HHBlits [13] for evolutionary related structures matching the target sequence. A total of 33 templates were found. Models were built based on the target-template alignment using Promod-II [14]. The global and per-residue model quality has been assessed using the QMEAN scoring function [15]. For improved performance, weights of the individual QMEAN terms have been trained specifically for SWISS-MODEL. The best model obtained from the server was further refined using the 3Drefine web-server [16]. 3D Refine uses a protocol consisting of two steps; a) optimization of hydrogen bonds network in the starting model, b) energy minimization. The first step involves a search for polar hydrogen atoms and their most favorable positions taking into account the hydrogen bonds formed with neighboring atoms based on the most probable protonation state for each amino acid. The output models of 3Drefine were analyzed for stereochemical properties using PROCHECK [17]; best quality model was chosen as the final model. Protein structures and models were visualized and rendered for figures using VMD [18].

### Identification of binding pockets

For identifying putative pockets in CPB, we used a consensus method by employing four binding pocket prediction algorithms, namely; CASTp, Fpocket, GHECOM and POCASA. Outputs from these algorithms were qualitatively analyzed to identify the most probable binding pockets. CASTp uses a weighted Delauneytriangulation method and the alpha-shape theory for shape measurements. Thereafter, it measures the area and volume of each pocket using analytical approaches, and also estimates the number of cavity opening, area of openings etc., in both Solvent Accessible Surface and Molecular Surface [19]. Similarly, Fpocket is also based on the concept of alpha spheres and Voronoi Tessellation for detecting alpha spheres - spheres that contact 4 atoms and do not contain internal atoms. These alpha spheres are clustered and analyzed to detect probable binding pockets in the protein [20]. GHECOM is a grid-based pocket detecting algorithm that utilizes the theory of mathematical morphology to define multi scale molecular volumes i.e. pockets defined using probes of different sizes [21]. POCASA implements an algorithm called Roll - a grid based pocket search method that uses a rolling spherical probe for pocket search. The pockets are subsequently ranked based on the cumulative distance of each pocket point from the pocket surface - highest scoring pockets are considered as ligand binding sites [22].

### Virtual screening of drug library:

Virtual screening was performed on CPB using a randomly selected subset of 5000 drug-like compounds obtained from the ZINC database [23]. Model for the CPB was prepared for virtual screening using AutoDockTools software. Particularly, polar hydrogens were added to the structure while non-polar hydrogens were merged with the aliphatic carbon atom, and appropriate partial charges were added. Similarly, the structures for each of the 5000 ligands were also prepared by adding Gasteiger-Marsili partial charges [24]. For virtual screening, a flexible docking approach was used wherein the receptor was represented as flexible in specific regions. Similarly, the ligands were also prepared so as to keep the rotatable bonds free during the docking process; such an approach allows for better sampling of the conformational space during the course of docking and has been shown to predict the ligand binding modes with greater accuracy. A large grid-box of size 100x126x126 grid-points was selected for specifying the docking search-space. The docking was performed using the Lamarckian Genetic Algorithm (GA-LS) implemented in AutoDock4.2 [25]. For each ligand, 20 runs of GA-LS were performed, each run starting with a population size of 200 and iterated for a number of generations until the maximum generation number (27000) or energy evaluation was reached (2000000),whichever is attained first. The best ligand compounds were selected based on their predicted binding-affinity values. The top 5 ligands were analyzed for their interactions to CPB using Chimera [26].

### Analysis of surface accessible cavities:

The protein structure was analyzed for buried cavities and channels using the CAVER algorithm as implemented in the software CAVER Analyst [27]. CAVER constructs a Voronoi map for a given structure consisting of vertices and edges. The tunnels and pathway through the protein are calculated by finding the shortest costs path between all surface vertices using Dijkstra's algorithm, and the final pathways are constructed by extending the paths to find a single low cost path to surface vertices.

## Results and Discussion:

**Figure 1:**
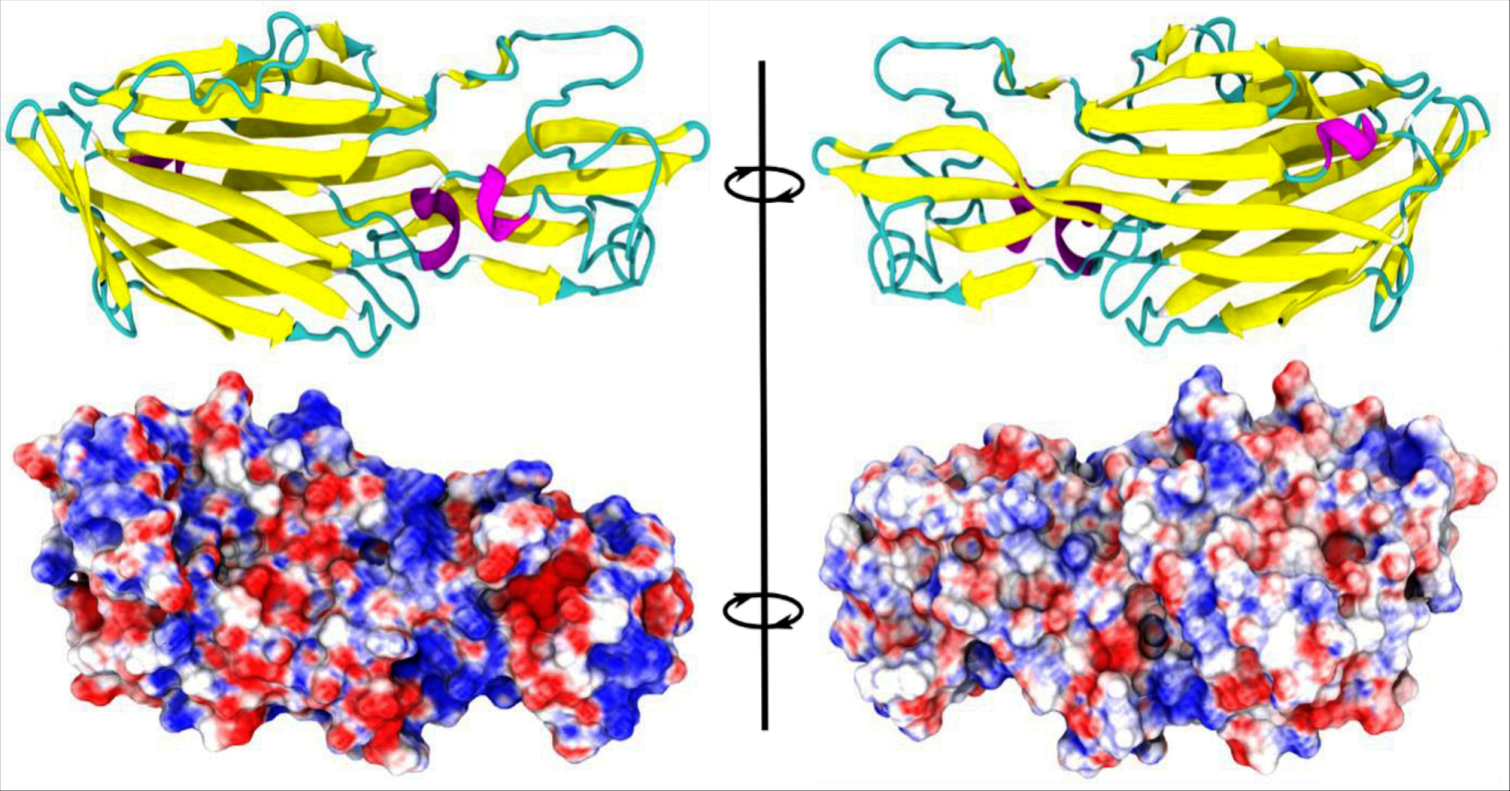
Homology model of Clostridium perfringens beta toxin (CPB). A model of CPB is depicted in ribbon representation. The lower panel is the surface representation of the toxin.

**Figure 2:**
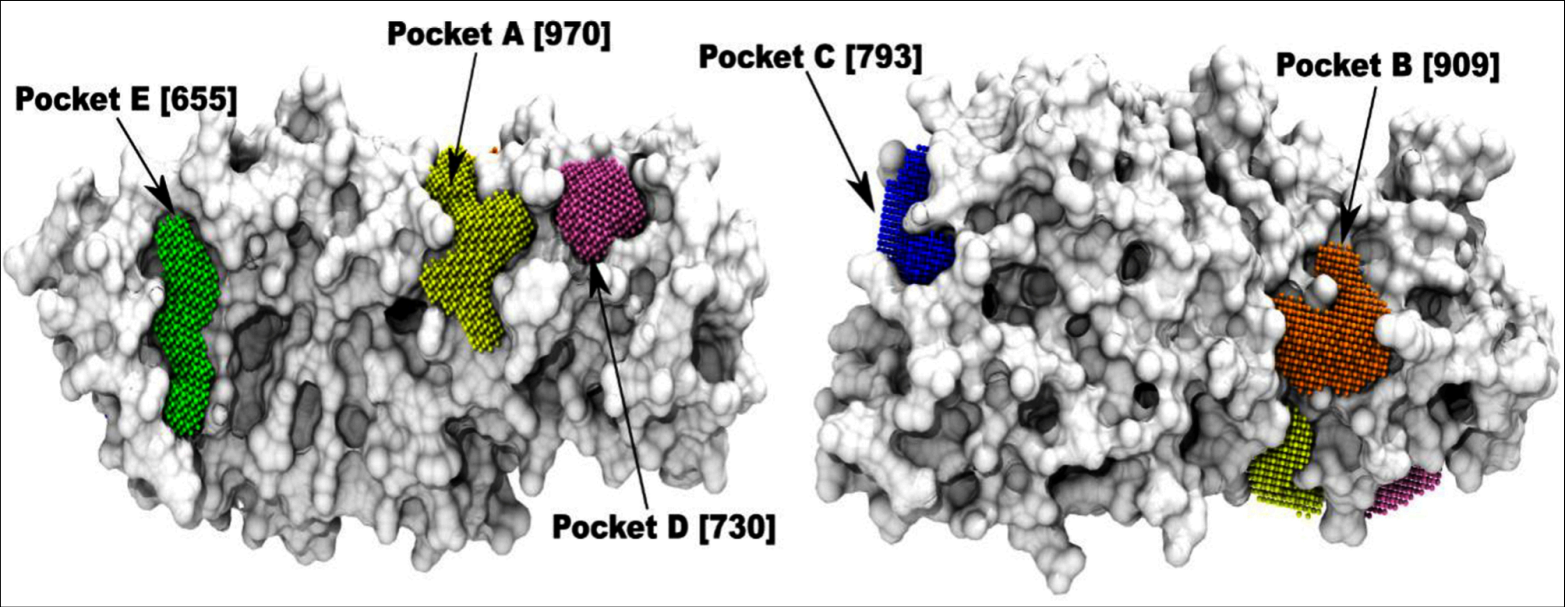
Prediction of putative binding pockets in CPB. Surface representation of CPB with the predicted top scoring pockets based on a consensus approach (see Materials and Methods). The predicted pockets named alphabetically in the order of decreasing pocket surface area (in brackets; Å^2^ units), are depicted as colored CPK points.

**Figure 3:**
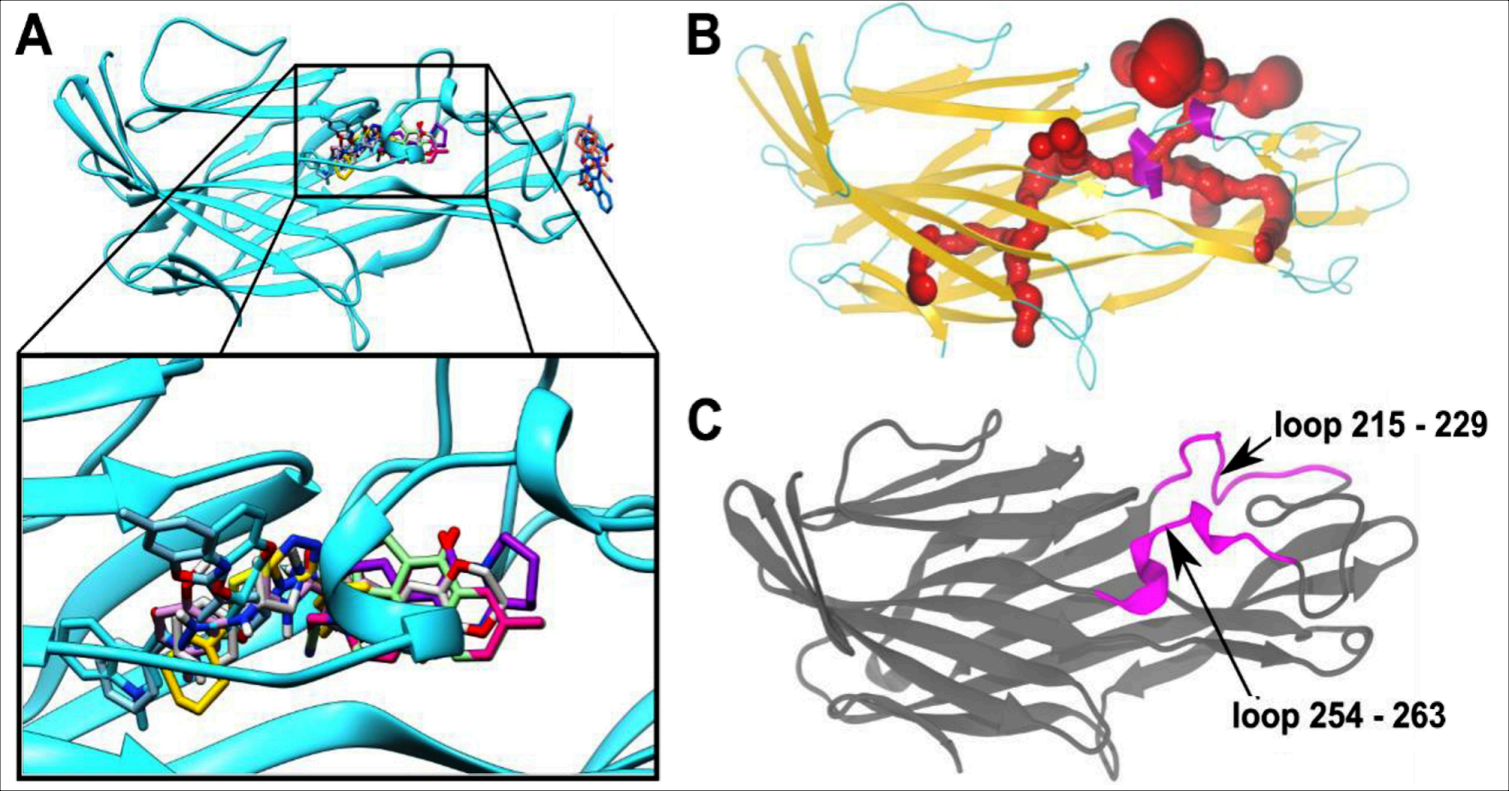
Docking studies and tunnel calculations on CPB. (A) Docking poses for the top 10 ligands predicted to bind CPB. The inset shows a zoomed view of Pocket A, shows a cluster of the top predicted binders; 8 out to the top 10 compounds are present in this cluster. (B) A model of CPB depicting the tunnels computed by CAVER, a program for computation of internal tunnels and cavities. (C) The top-ranking channel (depicted in red color in 3B) passed through the region between loops 215-229 and 254-263.

The structure of CPB from Clostridium perfringens is not available in the PDB database. Therefore, homology-modeling approach was employed to model the CPB structure. The model was generated by SWISS-MODEL webserver using the C. perfringens delta-toxin structure as template with a sequence identity of ~44%. The model generated from the server was further refined and subjected to stereo-chemical quality checks using PROCHECK; the model quality was found to be good with 99.6 % of the residues in allowed region and proper main-chain and side-chain parameters. However, the sequence coverage between the query and the template was around 86%; the first 27 residues of CPB could not be modeled. Modeling of the CPB using another program - Phyre2,[28] suggested that this N-terminal 27 residue region exists as an alpha-helical structure (extending from residue 5 to 27) that is connected to the rest of the protein via a 10-residue flexible linker region. The rest of the protein model predicted by Phyre2 (residues 28-336) was similar to the model obtained from SWISS-MODEL webserver with rmsd value of 0.636 Å. However, the N-terminal region was modeled at a very low confidence (>1%). Notably, in previous studies on CPB as well as delta-toxin, the first 27 residues segment has been suggested to act as a signal peptide directing the extracellular secretion of the toxin [29]. Therefore, the partial model (without the N-terminal segment) as generated using SWISSMODEL was selected for further structural investigations; the predicted model is depicted in Figure 1. The model of CPB is predominantly composed of beta-strands (arranged into anti-parallel beta-sheets) and loop regions. An interesting feature of the H-pore forming family of toxins is the presence of extensive loops within the structure; this points to an inherent flexibility in the loop regions and a wider conformational space. Specifically, in CPB the residues from 215 to 263 exist as a long coil region, which is stabilized by interaction with the underlying beta sheet. Such intrinsically flexible regions generally have implications to the functions of the protein. Pocket prediction methods were applied for the identification of such binding pockets in CPB. The output of four different pocket prediction methods to identify regions that are most likely to bind to a ligand was then analyzed. The top predicted pockets in CPB are illustrated in Figure 2. Out of these, Pocket A has the largest surface area of 970 Å^2^, followed by Pocket B with an area of 909 Å^2^. Pocket A is lined by part of the long loop segment (positions 215-263). A smaller pocket- D [730Å^2^] also lies in this region, lined by the loop residues. We therefore selected a large grid-box that incorporated the regions including the predicted pockets A, B and D (see Materials and Methods for details). The loop residues present within this grid-box were treated as flexible to account for protein flexibility while docking the ligands. The ligands used for docking were also treated as flexible by keeping all the rotatable bonds as free. The details of the top 10 docking results selected based on the predicted binding energies, are tabulated in Table 1(see PDF). Analysis of the docking poses of the top 10 ligands revealed that out of the 10 ligands 8 were docked in the region around Pocket A (Figure 3A). Structural analysis of these 8 docked conformations revealed that these ligands are significantly buried in the protein, accommodated within a cavity in the interior of the protein. Further analysis of CPB structure for surface accessible interior cavities and tunnels uncovered presence of cavities in the interior of the protein, especially in the region encompassing the long loop (Figure 3B). It is likely that the movement of the flexible loops in this region (highlighted in Figure 3C) allow accessibility to the buried cavities within the protein. Our docking protocol which treated these residues of the loops as flexible allowed us to simulate this flexibility and enabled identification of ligand binding modes that otherwise would be missed in a rigid body treatment. Apart from the 8 ligands that were docked in the same region of CPB, two other ligands (ligands 5 and 9 in Table 1 - see PDF) were docked to the peripheral region of CPB consisting of loops between H-strands (H6-H7, H15-H16) and loop residues 240-245 (part of the long loop 215-263).

**Figure 4:**
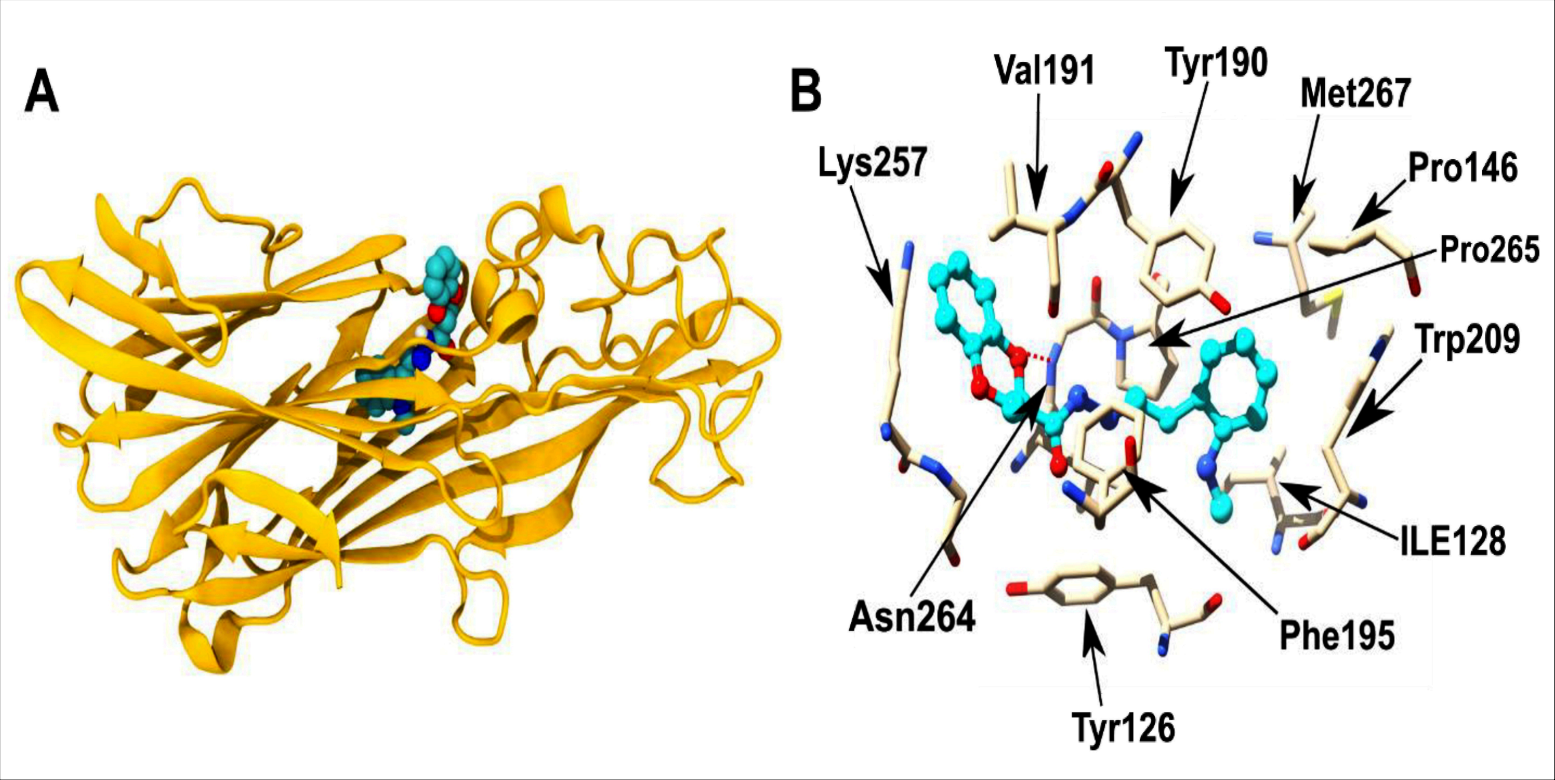
Binding pose for the top-scoring compound obtained from docking studies. (A) The top scoring compound - ZINC291192 [N-[(1-methylindol-3-yl)methyleneamino]-7,10-dioxabicyclo[4.4.0]deca-2,4,11-triene-8-carboxamide] (depicted in CPK representation) bound to CPB. (B) The residues interacting with the top scoring compound (in ball and stick; cyan color). H-bond between the compound and Asn264 is depicted as red dotted line.

The best scoring binding-model of the top inhibitor ZINC291192 [N-[(1-methylindol-3-yl)methyleneamino]-7,10-dioxabicyclo[4.4.0]deca-2,4,11-triene-8-carboxamide] is depicted in Figure 4. This inhibitor was predicted to have a binding energy of -10.38 kcal/mol that corresponds to an estimated dissociation constant of 2.4 x 10^-8^ M. The residues that make up the pocket that binds to the inhibitor are mostly composed of hydrophobic residues indicating that the inhibitor is largely involved in hydrophobic and van der Waals forces. Besides, the inhibitor forms a hydrogen bond interaction with the backbone of Asn264 residue. These interactions together with a significant desolvation energy, due to the transfer of the inhibitor compound from solvent to the hydrophobic pocket, results in the high estimated binding energy. Thus, in the present study we have identified a potential inhibitor for CPB secreted by C. perfringens type C. Since the C. perfringens type C is not affected by antibiotic treatments and is capable of causing necrotizing enteritis independently, it is necessary to identify inhibitors of CPB for which a structure based in silico screening of compounds was performed using ZINC database. The best hit compound ZINC291192 had a predicted binding energy of -10.38 kcal/mol corresponding to an estimated dissociation constant of 2.4x 10^-8^ M, which can be studied further for clinical applications. For a more thorough investigation, the top 5 molecules binding at the aforementioned region (corresponding to pocket A) can also be tested for their inhibitory potential; the top 5 inhibitors have an estimated dissociation constant ranging from 2.4 x 10^-8^ to 8.1 x 10^-8^ M which indicates a significant binding affinity for these ligands for further consideration in validation.

## Conclusion:

We report a potential inhibitor for beta toxin from Clostridium perfringens from ZINC database with predicted binding features such as binding energy of -10.38 kcal/mol corresponding to an estimated dissociation constant of 2.4 x 10^-8^ M for further consideration in drug discovery.
